# The optimization and application of photodynamic diagnosis and autofluorescence imaging in tumor diagnosis and guided surgery: current status and future prospects

**DOI:** 10.3389/fonc.2024.1503404

**Published:** 2025-01-08

**Authors:** Wei Wan, Huiquan Liu, Junrong Zou, Tianpeng Xie, Guoxi Zhang, Weihai Ying, Xiaofeng Zou

**Affiliations:** ^1^ The First Clinical College, Gannan Medical University, Ganzhou, Jiangxi, China; ^2^ Institute of Urology, The First Affiliated Hospital of Gannan Medical University, Ganzhou, China; ^3^ Department of Urology, The First Affiliated Hospital of Gannan Medical University, Ganzhou, China; ^4^ School of Biomedical Engineering and Med-X Research Institute, Shanghai Jiao Tong University, Shanghai, China

**Keywords:** photodynamic diagnosis, autofluorescence imaging, photosensitizer, tumor, fluorescence-guided surgery, diagnosis, optimization strategy

## Abstract

Photodynamic diagnosis (PDD) and autofluorescence imaging (AFI) are emerging cancer diagnostic technologies that offer significant advantages over traditional white-light endoscopy in detecting precancerous lesions and early-stage cancers; moreover, they hold promising potential in fluorescence-guided surgery (FGS) for tumors. However, their shortcomings have somewhat hindered the clinical application of PDD and AFI. Therefore, it is imperative to enhance the efficacy of PDD and AFI, thereby maximizing their potential for practical clinical use. This article reviews the principles, characteristics, current research status, and advancements of PDD and AFI, focusing on analyzing and discussing the optimization strategies of PDD and AFI in tumor diagnosis and FGS scenarios. Considering the practical and technical feasibility, optimizing PDD and AFI may result in an effective real-time diagnostic tool to guide clinicians in tumor diagnosis and surgical guidance to achieve the best results.

## 1 Introduction

Cancer poses a serious threat to global human health and has surpassed cardiovascular diseases as the leading cause of death in economically developed countries while remaining a major cause of mortality in developing countries (1). According to the estimates of GLOBOCAN (2), approximately 20 million new cases of cancer were recorded in 2022, with nearly 9.7 million cancer-related deaths. Considering the growing incidence and mortality rates of cancer, improving cancer detection to achieve early diagnosis and treatment plays a crucial role in enhancing the prognosis of cancer patients. Fluorescence imaging techniques, such as photodynamic diagnosis (PDD) and autofluorescence imaging (AFI), have demonstrated significant advantages and potential in the early detection of cancer (3, 4).

Over the past few decades, significant advancements have been made in the application of fluorescence imaging technology in cancer diagnosis, particularly in the field of gastrointestinal tumor imaging, as well as in the diagnosis of tumors in the respiratory tract, skin, and bladder (5–8). Currently, fluorescence imaging plays a pivotal role in tumor diagnosis, surgical guidance, and intraoperative margin assessment, especially in the early diagnosis of cancer and intraoperative precision localization, thereby improving patient prognosis (9, 10). In traditional cancer surgery, decisions are mainly based on imaging data, the visual appearance of the tumor, and palpation. In contrast, PDD and AFI provide real-time, convenient, and accurate fluorescent image guidance for surgery (4, 6). Traditional endoscopy can only detect lesions based on the overall morphological changes, which is prone to missed diagnosis (11). However, PDD and AFI can identify precancerous changes and early tumors by examining the microstructural, biochemical, and molecular characteristics.

In addition, The ideal optical diagnostic techniques are non-invasive, objective, and reusable, offering high diagnostic accuracy and low toxicity. Nonetheless, no single optical imaging technique incorporates all these properties. Currently, optimizing or refining detection techniques has become a routine practice in the process of cancer diagnosis and surgical excision (12). Therefore, the optimization of PDD and AFI is of great clinical significance for improving the diagnosis and surgical guidance of traditional endoscopy in tumors.

## 2 Photodynamic diagnosis

### 2.1 PDD principles and characteristics

**Figure 1:**
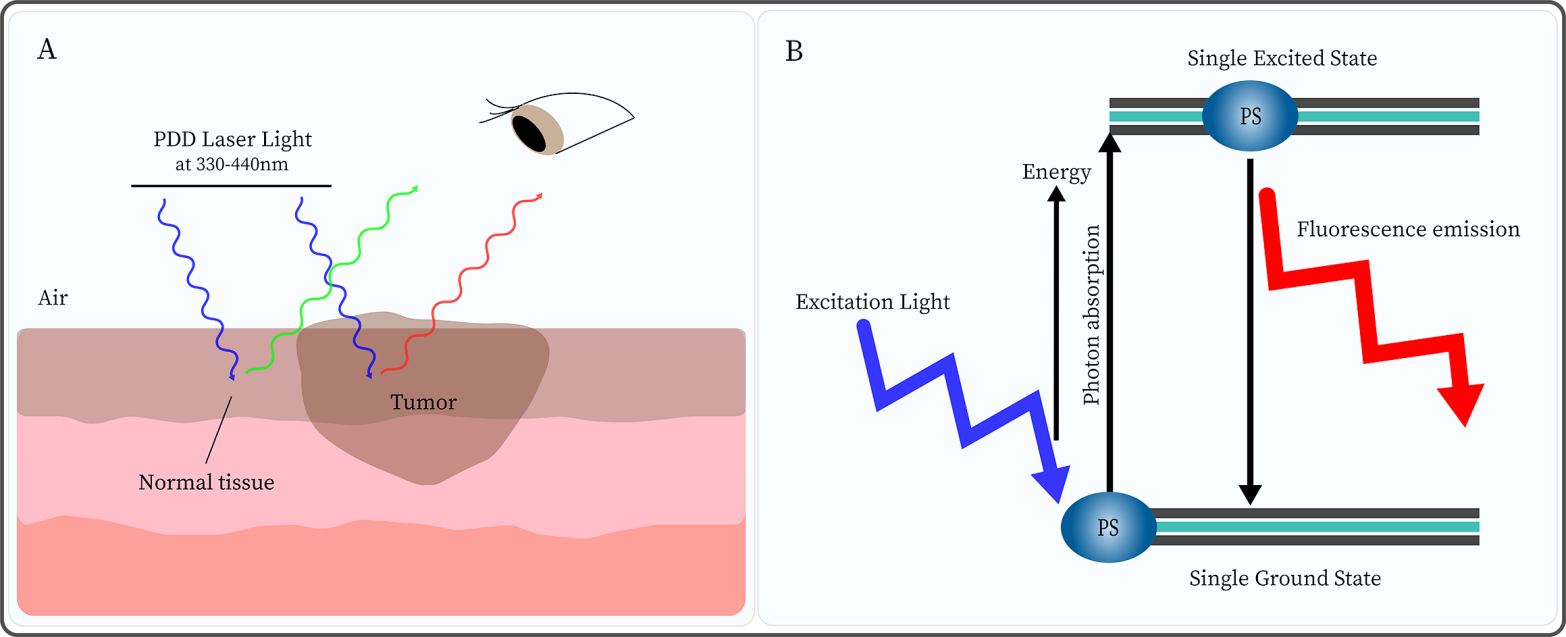
Photodynamic diagnosis (PDD) is based on the different concentrations of photosensitizer (PS) in normal and tumor tissues. **(A)** After injection of PS, under PDD laser light (330-440nm) irradiation, normal tissue emits green fluorescence while tumor tissue displays red fluorescence. **(B)** Under excitation light irradiation, the PS molecule absorbs energy from the ground state to the single excited state. Subsequently, the PS returns to the ground state while emitting light of a higher wavelength (lower energy) than that used for excitation.

Photodynamic diagnosis (PDD), also known as fluorescence endoscopy, is an optical diagnostic technique that relies on an exogenous probe, such as a photosensitizer (PS), as a contrast mechanism to indicate pathological tissue (13). PS is injected, which is selectively accumulated in cancer cells, and exposure to excitation light of a certain wavelength triggers a unique fluorescence (14) (**Figure 1**). Thus, the fluorescence produced by PS differentiates between normal and abnormal tissue (14).

Notably, PDD uses a PS that has a high affinity for tumor tissue, is highly specific, and is primarily excited in the lower visible blue wavelength range. Still, the activation of PS in the excited singlet state is transient, with a lifespan typically ranging from a few nanoseconds to a few picoseconds. This process does not induce cell signal death pathways, and can only be used for fluorescence diagnosis, without causing cell damage (15). Moreover, PDD can be used to visualize biological tissues and identify disease areas. Moreover, multiple clinical trials of PDD have shown that its tumor-detection capabilities exceed that of white-light endoscopy (WLE) (16, 17), which greatly improves the early diagnosis of tumors and the prognosis.

### 2.2 The application and shortcomings of PDD

Currently, PDD technology is widely applied in the diagnosis of clinical tumors and fluorescence-guided surgery (FGS) (18, 19). PS plays an essential role in the PDD process. The compound selectively accumulates in tumor cells and is activated by light of a specific wavelength to trigger fluorescence, thereby promoting effective PDD (20). In the PDD of tumors, the most commonly used PSs (mainly second-generation) include 5-aminolevulinic acid (5-ALA) (21), hexaminolevulinate (HAL) (22), methylene blue (23), and indocyanine green (ICG) (24), etc. The classification and characteristics of PS are shown in **Table 1**.

**Table 1:** Characteristics of different generations of PSs.

PS generation	Biological characteristics	PS Example	PDD (exc)/(em)
1st PS* (Low chemical purity and stability)	Only passive uptake Low of light absorption and intracellular localization	Hematoporphyrin	(330-410 nm) / (630 nm)
2nd PS (Overall performance is better than 1st PS)	Shorter half-life Improved passive uptake and tissue light penetration Minimized adverse reactions Poor water solubility and sub-cellular localization	5-ALA HAL ICG MB	(375-490 nm) / (600-740 nm) (360-465 nm) / (610-650 nm) (780 nm) / (820-850 nm) (670 nm) / (700 nm)
3rd PS (2nd PS + Nanoparticles + Target biomolecules)	Deeper tissue light penetration Improved PS tumor cell specific uptake and localization	ZnPcS4 + AuNP + Anti-GCC mAb	(330-350 nm) / (620-640 nm)

*PS, Photosensitizer; 5-ALA, 5-Aminolevulinic acid; HAL, Hexaminolevulinate; ICG, Indocyanine green; MB, Methylene blue; ZnPcS4, Zinc sulfothiolphthalocyanine; AuNP, Gold nanoparticle.

5-ALA is the most extensively studied photosensitizer to date and has been approved by the European Union since 2007 for use in fluorescence-guided resection of malignant gliomas (25). However, in a systematic retrospective analysis of FGS applications to 467 low-grade gliomas, the results showed that fluorescence positivity was found in 34 out of 451 (7.3%) of grade II tumors in 5-ALA-mediated PDD; whereas in 9 out of 16 (56.2%) of grade I tumors, the mean fluorescence rate of 5-ALA was 9.2% (26). Due to lower fluorescence rates and limited local bioavailability (e.g., poor lipophilicity (27)) hinders the use of 5-ALA in daily clinical practice. Therefore, a more lipophilic PS was synthesized. Compared with 5-ALA, HAL exhibits a higher efficiency in converting to protoporphyrin IX (PpIX), stronger tissue penetration, and a higher PpIX fluorescence intensity at lower concentrations (28). In a study of 699 nonmuscle invasive bladder cancer patients, Drejer et al. (29) observed tumor recurrence within 8 months after randomization under HAL-mediated PDD and WLE examinations. Results showed 117 of 351 patients in the PDD intervention group had recurrence, while 143 of 348 in the WLE control group (*P*=0.049), with an odds ratio of 0.67 (*P*=0.02, 95% CI: 0.48-0.95). Moreover, Lapini et al. (30) The diagnostic accuracy of PDD versus WLE in bladder tumors was compared and the results showed that WLE guided biopsy had a sensitivity of 76.8%, a specificity of 36.5%, a positive predictive value of 50.9%, and a negative predictive value of 64.8%, while HAL-PDD guided biopsy had a sensitivity of 99.1% (significantly higher than that of WLE, *p*<0.00001), specificity 30.2% (not significantly different from WLE), positive predictive value 54.9%, and negative predictive value 97.4%. The proportion of patients correctly diagnosed with PDD and WLE was 97.9% and 88.5%, respectively (*p*=0.0265). This further indicated that HAL-mediated PDD has a higher detection capability for precancerous lesions and early-stage tumors compared to traditional WLE. However, the HAL excitation range falls within the visible light spectrum, and the compound has poor tissue penetration. In contrast, ICG, as a second-generation photosensitizer excited at near-infrared wavelengths, is characterized by safety, simplicity, rapidity, and avoidance of autofluorescence interference, which has facilitated its widespread application (31–33). Shiomi et al. (34) and Bargon et al. (24) found that ICG-mediated PDD had a positive role in detecting and guiding the localization of intraoperative sentinel lymph nodes in esophageal cancer and breast cancer, respectively. Nevertheless, the clinical application of ICG-mediated PDD in tumor diagnosis and FGS still confronts several challenges. For instance, ICG exhibits a low photostability, a moderate fluorescence quantum yield, and a high plasma protein binding rate (35). Simultaneously, the *in vivo* fluorescence duration of ICG is brief. This is because free ICG has a propensity to aggregate rapidly and be eliminated from the body within the physiological environment (36), and it also demonstrates a low specificity for target tumor cells (37).

**Figure 2:**
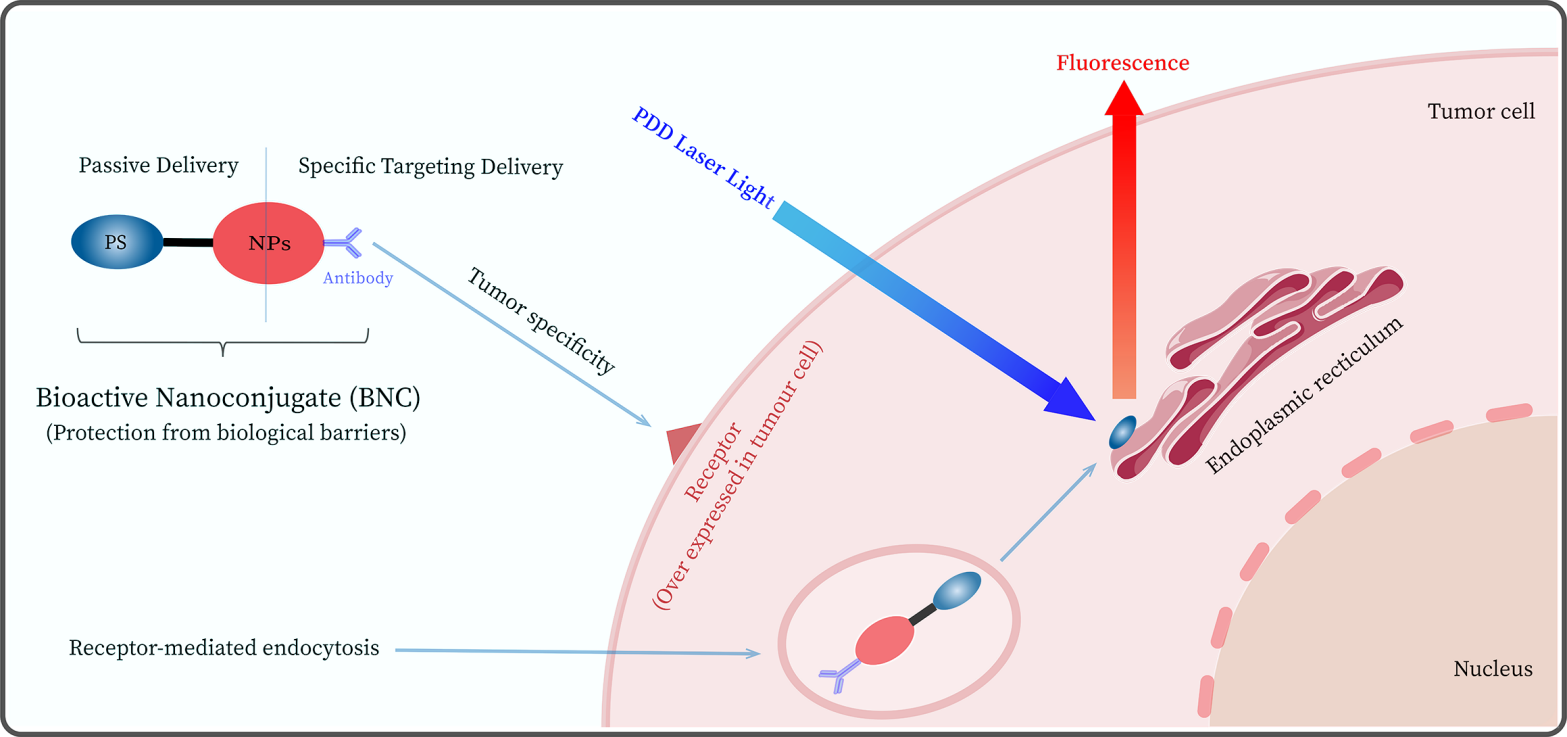
Schematic diagram of the photodynamic diagnosis PDD mediated by a bioactive nanoconjugate (BNC) composed of nanoparticles (NPs), antibody, and photosensitizer.

Currently, researchers are attempting to overcome the shortcomings of second-generation photosensitizers by developing probes that can specifically recognize and target tumor cells (e.g., third-generation PSs), ensuring selective recognition and enhanced diagnostic efficacy (38). Nanoparticles (NPs) have been employed with great success as passive carriers (39) for the study of third-generation PSs, such as gold nanoparticles (AuNPs), silver nanoparticles, polymer nanoparticles, and silicon-based materials (40, 41). In addition, NPs can achieve passive targeting of tumors through enhanced permeability and retention (EPR) effects. The EPR passive uptake effect allows NPs-mediated PS to move freely into the tumor microvascular system through porous blood vessels and lymphatic drainage, thereby increasing PS localization in tumor cells (42). The advantages of bioactive nanoconjugates (BNCs) of NPs bound to PSs lie in their unique physicochemical properties (e.g., EPR effect) and the ability to improve the selectivity and specific targeting of second-generation PS through chemical modification (43–45). The BNC-mediated PDD process is displayed in **Figure 2**.

In addition, the target cell specificity of BNCs can be significantly improved by modifying the surface of BNC to bind active targeting fractions, such as antibodies (46). For example, Deken’s group (47) investigated the *in vitro* and *in vivo* antitumor efficacy of photodynamic diagnosis and therapy using BNCs targeting HER2, which were found to bind specifically to the target and showed a higher affinity for HER2-positive tumor cells. Although BNC-mediated PDD has many advantages and potential for clinical applications. However, it faces challenges in mediating fluorescent diagnostics and surgical navigation, such as *in vivo* environmental interference, non-specific binding, altered photophysical and chemical properties, PS drug release, and internalization issues (48–50). Moreover, PSs may have negative effects, such as pain, vomiting, and hypotension (51, 52), which limits their clinical application to a certain extent. Therefore, there is a need to further optimize BNC-mediated PDD (Such as reducing PS side effects and improving BNC optical stability, etc.) to improve its shortcomings in diagnostic and FGS application scenarios.

## 3 Autofluorescence imaging

### 3.1 AFI principles and characteristics

**Figure 3:**
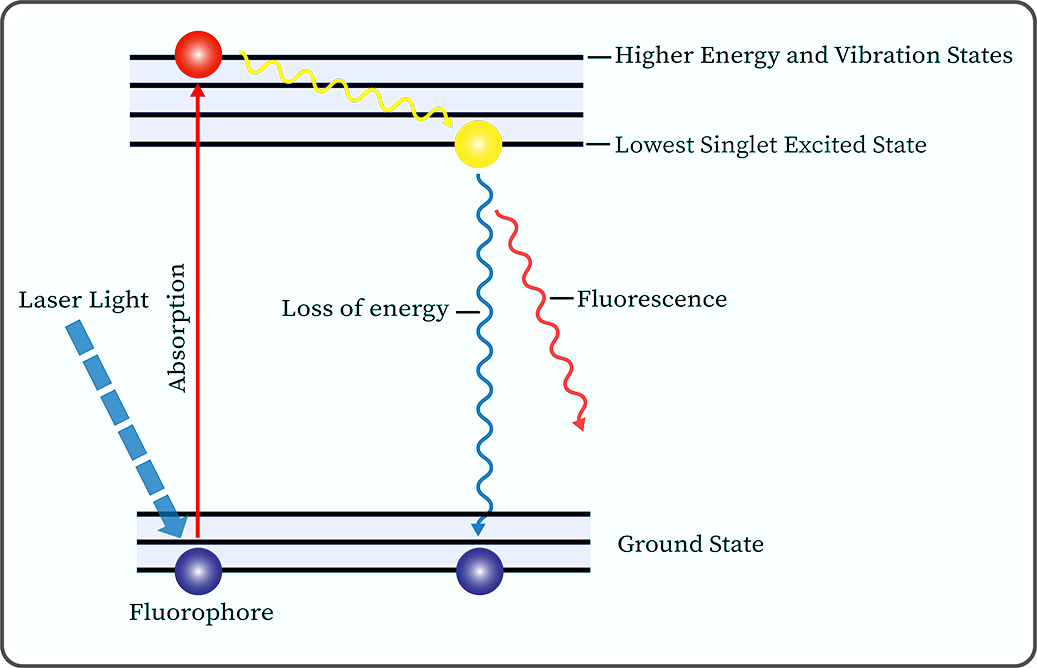
Jablonski diagram illustrates the molecular mechanism of autofluorescence phenomenon produced by fluorophores under laser light irradiation.

Autofluorescence (AF) is a widespread phenomenon in plants and animals. Cells and matrices in biological tissues contain many molecules, such as NADH, FAD, elastin, and porphyrin (53–56), which can produce AF signals corresponding to their absorption spectra when irradiated with excitation light of certain wavelengths (**Figure 3**).

At the beginning of the last century, scientists discovered that excised tumor tissues can emit fluorescence under ultraviolet light irradiation. Subsequently, biologists have conducted extensive research on various fluorophores within organisms, revealing that the fluorescence characteristics of these molecules are related to their physicochemical properties (57, 58). Autofluorescence imaging (AFI), based on the principle of AF, involves the emission of longer wavelength AF signals by endogenous fluorophores in biological tissues under the irradiation of excitation light at specific wavelengths. These signals are then collected and processed by special sensors, which display unique fluorescent images or spectra (59). It is noteworthy that for different types of cancer, the AF intensity of cancerous tissues can be either higher or lower than that of normal tissues, e.g., melanoma’s AF is higher than that in normal tissues (60), while our team’s previous study found that the green AF intensity of the cancerous tissues in the lung parenchyma of lung cancer patients was significantly lower than that of the normal tissues of the lung cancer patients (61). Our study suggested that the significantly decreased green AF intensity, which may result from changes of the AF of keratins, can become a potential biomarker for non-invasive diagnosis of lung cancer (61). Therefore, normal tissues can be distinguished from cancerous tissues based on the differences in the AF images or spectral signals of the tissues to achieve the purpose of clinical diagnosis.

### 3.2 The application and shortcomings of AFI

AFI does not rely on exogenous probes (e.g., ICG (62)) for labeling and does not require the removal of tissue specimens for testing (63). Therefore, this method avoids liver and kidney function damage and many adverse reactions caused by exogenous tracers. In addition, the superior sensitivity of AFI compared to WLE may be related to its ability to detect subtle fluorescence changes produced by precancerous lesions or early tumors. Differences in overall fluorescence emission between normal and abnormal tissues are due to differences in fluorescent molecule concentration, metabolic state, and spatial distribution (64). And this difference in color or intensity of fluorescence emission can be captured in real-time in the AF assay for early microscopic lesions not detected by WLE.

AFI endoscopy (AFIE), which combines AFI technology with a number of endoscopes commonly used in clinical practice, has greatly improved the diagnosis of precancerous lesions and early tumors (11). Early AFIEs were mainly fiber-optic endoscopes, which diagnosed pre-cancerous lesions and early tumors by detecting real-time pseudo-color images generated by AF of their own tissues. For example, Niepsuj et al. further tested the detection capability of AFIE in a study of 34 patients with short-segment Barrett’s esophagus. Under AFIE and WLE examination, 109 and 136 biopsy specimens were removed from Barrett’s mucosa, respectively, and it was found that the frequency of detection in AFIE-guided biopsy specimens was significantly higher than in WLE-guided biopsy specimens (8.3% vs. 0.7%; *p*=0.016) (65). Moreover, Sun et al. (66) conducted a meta-analysis of AF and white light bronchoscopy (WLB) for detecting bronchial carcinoma. Ten articles involving 1,830 patients’ data were included. The results showed that the sensitivity of AFI was 0.92 (95% CI: 0.88-0.95), which was higher than that of WLB 0.70 (95% CI: 0.58-0.80, *p*<0.01). The specificity of AFI was 0.67 (95% CI: 0.51-0.80), while that of WLB was 0.78 (95% CI: 0.68-0.86, *p*=0.056). The positive predictive values of AFI vs. WLB were 85.0% and 76.7% respectively (*p*<0.05), and the negative predictive values of AFI vs. WLB were 67.6% and 70.5% respectively (*p*=0.06). The area under the curve (AUC) of AFI was 0.92, and that of WLB was 0.81. The Egger test yielded a P value of 0.225, indicating no publication bias. These studies demonstrated that AFI detects lesions better than WLB and has a higher sensitivity. In addition, Moriichi’s team demonstrated the potential of the AFI system for the detection of precancerous lesions and early tumors in a study evaluating the grading of heterogeneous hyperplasia in colon tumors (67). Subsequently, Takeuchi et al. (68) further tested the detection ability of AFIE through a multicenter randomized controlled trial. Patients were randomly divided into the WLE group (404 patients) and the AFIE group (398 patients). The results showed that the number of flat tumors detected in the AFIE group was significantly higher than that in the WLE group (0.87 (95% CI, 0.78-0.97) vs. 0.53 (95% CI, 0.46-0.61)). In another visualization study using AFI video-endoscopy versus WLE to assess squamous cell carcinoma of the esophagus and pharynx, Suzuki et al. found that the proportion of lesions that were clearly visible on AFI video-endoscopy was significantly higher than on WLE (79% vs. 51%; *p*<0.05) (69). These studies demonstrated the potential of AFI for the clinical diagnosis of precancerous lesions and early tumors. Furthermore, AFI has shown great potential in the application of FGS (70). For instance, Thomas et al. (71) achieved enhanced intraoperative adrenal visualization and effective tumor resection during adrenalectomy by introducing near-infrared AFI detection technology during surgery. In another study on intraoperative AF detection of parathyroid glands that lasted 5 years, Ladurner et al. (72) used AFI as a detection method during surgery and examined a total of 205 parathyroid glands in 117 patients. Among these, 179 glands (87.3%) were correctly identified by AF.

Nonetheless, the clinical application of AFI is circumscribed. In the current era of high-definition video endoscopes, the image quality associated with AFIE based on fiber-optic technology is considerably subpar (the fiber-optic technology provides relatively low resolution and contrast (73)), and the cumbersome imaging platform system gives rise to its unsatisfactory operability (68, 73). Meanwhile, the specificity of AFI diagnosis is relatively low, with a relatively high false positive rate; the equipment cost of AFI is higher than that of conventional WLE, and the image quality is easily disturbed by several factors (such as the internal and external environments of tissue cells, the imaging environment, the performance of signal sensors and operation techniques, etc.) (74–77). To some extent, these drawbacks limit the clinical application and adoption of AFI systems. Thus, AFI should be further optimized to improve its deficiencies in diagnostic and FGS application scenarios, which is of positive significance for improving the prognosis of cancer patients.

## 4 Optimization strategy for PDD and AFI

### 4.1 Optimization in diagnosis

Effective PDD relies on exogenous probes, selecting PS with known photophysical and pharmacokinetic properties. Additionally, exogenous PS exhibits stronger fluorescence than endogenous fluorophores. However, second-generation PSs have lower specificity (78), have significant side effects (79), and increase the cost of medication, thereby limiting the use of PDD in tumor diagnosis. In addition, PS needs to be injected before the start of PDD, and the long interval between PS administration and irradiation greatly extends the duration of diagnosis and treatment, while reducing the comfort of the therapy (80). Moreover, the dosage and method of administration for PS have not been standardized, potentially leading to excessive or insufficient dosages of PS (81). Therefore, PDD should be further optimized. For example, BNC (third-generation PSs) can reduce the adverse effects of PDD, thereby enhancing its potential for clinical application (82). Simelane’s group (83) successfully prepared a BNC based on polyethylene glycolated AuNP and showed selectively improved subcellular accumulation within the target colorectal cancer, somewhat optimizing the drawbacks of insufficient PDD specificity.

Furthermore, regardless of how PS interacts with the target tissue, the continuous emission of its fluorescent signal will ultimately generate a certain level of background signal interference (84). In contrast, activatable fluorescent probes are optically silent and emit strong fluorescence only in tumors (e.g., AVB-620 (85)). Therefore, employing an activatable PS reduces the interference of background signals and improves the diagnostic ability of PDD. Nonetheless, PDD-based FGS is still limited to clinical trials due to the shortcomings of PS drugs. However, these can be circumvented by AFI, which does not rely on exogenous probes for tumor detection. Thus, the rapid metabolic clearance of PS *in vivo* as well as its adverse outcomes for patients can be significantly improved by combining PDD with AFI.

The diagnostic fundamentals of AFI and PDD are similar. The main difference is that the former does not rely on exogenous PS (**Table 2**), which greatly avoids unnecessary waiting times and the toxic side effects of PS. Szygula’s group (86) compared the sensitivity and specificity of AFI versus PDD in the diagnosis of bladder tumors, revealing that the sensitivity and specificity of PDD were 90.91% and 66.60%, as opposed to 97.83% and 70.07% for AFI, respectively. These results demonstrate that AFI offers a more sensitive diagnosis of intravesical lesions than PDD (AFI vs. PDD; *p*=0.0056). However, the findings of Kriegmair et al. (70) and Kuiper et al. (87) revealed that AFI had low specificity in the diagnosis of flat tumors. This may be attributed to the differences in AFI images or spectra being affected by the internal and external environment of tissue cells (such as the concentration of fluorophores, mucosal thickness, blood concentration, etc.) and their absorption and scattering of light (76, 88). Therefore, AFI requires further optimization to minimize the impact of hemoglobin absorption spectra (89).

**Table 2:** Characteristics of autofluorescence imaging and photodynamic diagnosis.

Technology	Principle	Fluorescent probe	Advantages and Disadvantages	Application scenario
AFI*	Tissue endogenous fluorophores can produce Autofluorescence signals corresponding to their absorption spectra when irradiated with a certain wavelength of excitation light	NADH FAD Collagen Keratin PpIX and Porphyrins	Advantages: Real-time, rapid, without PS, economical and prevent the negative effects of non–uniform distributions of the probes Disadvantages: Low fluorescence intensity, poor specificity and tissue penetration	Diagnosis / Fluorescence Guided Surgery
PDD	This optical diagnostic technique employs PS as a contrast mechanism for the identification of pathological tissue	5-ALA HAL ICG BNC	Advantages: High specificity, clear imaging and deep tissue penetration Disadvantages: Time-consuming, poisonous side effect and rapid clearance of PS	Diagnosis / Fluorescence Guided Surgery

*AFI, Autofluorescence imaging; PDD, Photodynamic diagnosis; PS, Photosensitizer; 5-ALA, 5-Aminolevulinic acid; HAL, Hexaminolevulinate; ICG, indocyanine green; NADH, Nicotinamide adenine dinucleotide; FAD, Flavin adenine dinucleotide; PpIX, Porphyrins IX; BNC, Bioactive Nanoconjugate.

Moreover, AFI is not as effective as narrowband imaging (NBI) (90) in observing submucosal blood vessels and mucosal morphology, thus combining with NBI may further improve the ability of AFI in tumor diagnosis (91). Additionally, fluorescence intensity is an important parameter in tumor diagnosis with AFI or PDD. However, the fluorescence emission spectra of the fluorophores or PS of interest often overlap with those of other fluorophores in the body, which poses a certain difficulty in distinguishing between them. In contrast, fluorescence lifetime measurements utilize not only the fluorescence intensity of the fluorophore but also its fluorescence lifetime (92). Compared to fluorescence intensity measurements, fluorescence lifetime measurements provide additional information about the sample and facilitate the differentiation between scattered light and potentially endogenous fluorophores (93), and often avoid some of the factors that affect fluorescence intensity measurements (such as photobleaching, concentration, wavelength, etc.) (94). Therefore, combining fluorescence lifetime measurement technology can further optimize the fluorescence diagnostic capabilities of PDD and AFI. In the past several years, artificial intelligence (AI) technology has made profound impact on clinical medicine (95), including fluorescence-based diagnostics (96). Currently, AI technologies are widely used to enhance fluorescence imaging, process large amounts of complex and abstract data, perform pattern recognition and image analysis, and provide intelligent diagnosis (95, 97). This somewhat ameliorates the poor imaging quality due to the inadequacy of fluorescent probes. Therefore, a combined strategy with AI may overcome technical barriers and improve the diagnostic accuracy of AFI with PDD to meet the requirements of precision cancer surgery.

### 4.2 Optimization in FGS

**Figure 4:**
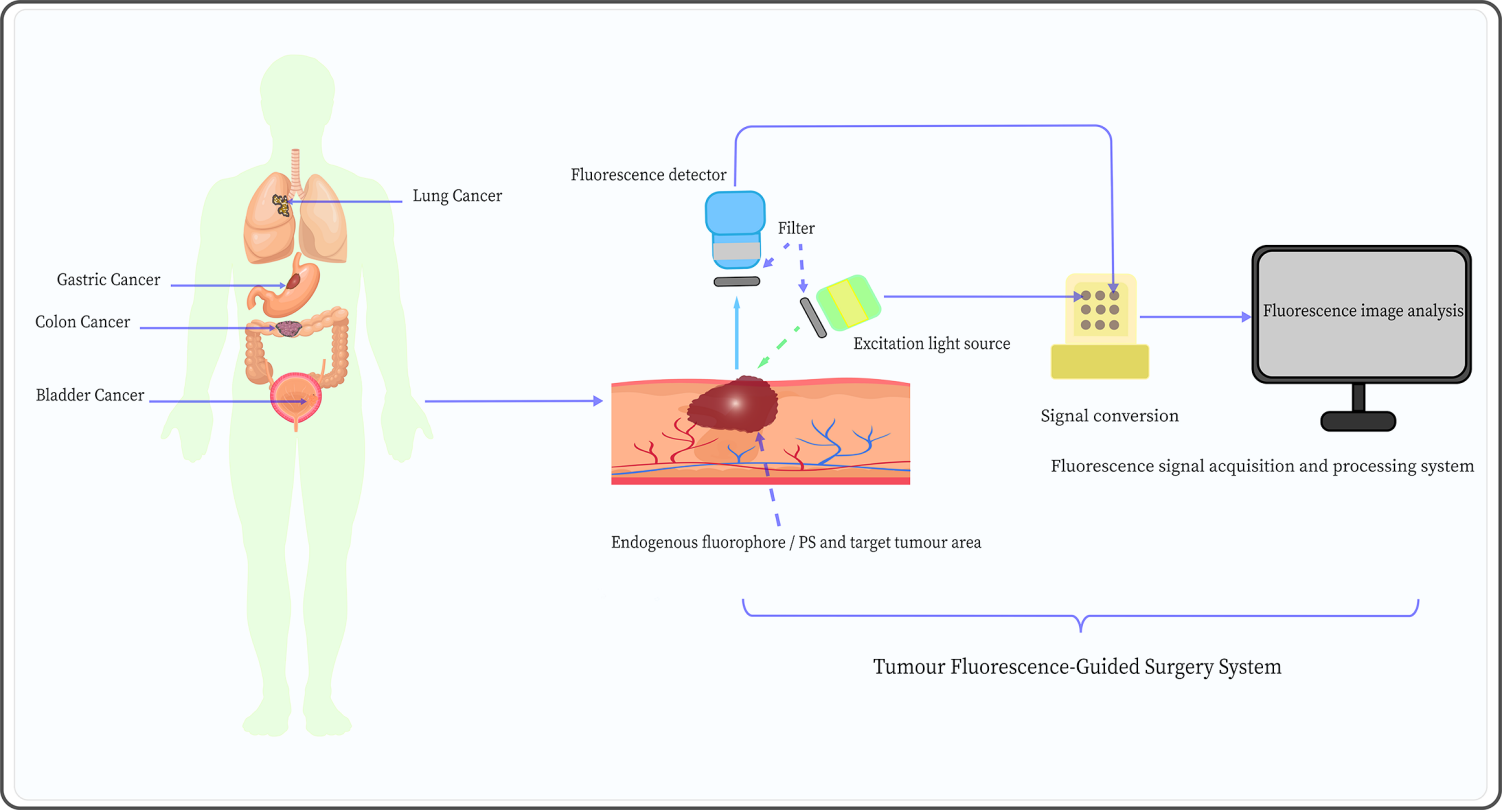
Schematic diagram of the tumor fluorescence-guided surgery system mediated by AFI and PDD, including an excitation light source, appropriate fluorescence detector, and fluorescence signal acquisition and processing system.

The FGS system employing PDD and AFI mainly consists of three components: the excitation light source, the signal detector, and the signal acquisition and processing system, as shown in **Figure 4**. These fluorescence-related components are primarily based on mature technologies and methods that have been widely used in fluorescence spectroscopy and microscopy (98). However, the components of the fluorescence imaging system have some shortcomings that limit their application in FGS. Therefore, each fluorescence imaging system component should be further optimized to enhance the application of PDD and AFI in FGS.

Common excitation light sources for PDD and AFI include traditional light sources (mercury arc lamp systems) and light-emitting diodes (LEDs). Compared to traditional light sources, LEDs are characterized by longer lifespan, narrower bandwidth emission (typically 20-30 nm), and higher signal-to-noise ratio (99). However, the narrower bandwidth implies that LEDs are not suitable for use with fluorophores with small Stokes shifts. The laser light sources have high selectivity in wavelength, which is typically much more intense; even if the wavelength does not perfectly match that of the target fluorophore, these light sources can still trigger fluorescence, such as laser diodes (100) and super-continuum laser sources (101). Nevertheless, the size and cost of laser light sources are higher than those of LEDs, which limits their application in FGS. Therefore, new laser light sources should be optimized to overcome their bulky size, simplify systems, and decrease cost in the application of PDD and AFI in FGS.

Fluorescence signal detectors are essential components of fluorescence imaging systems. Photomultiplier tubes (PMTs) are the most commonly used non-photon number-resolving detectors in biology and medicine and are characterized by high gain, low noise, fast response, and low-temperature sensitivity (102). Nonetheless, PMTs are bulky in size, require a high-voltage power supply, and are sensitive to magnetic fields (77). In contrast, silicon photomultipliers (SiPMs) have the advantages of small size, low energy and bias voltage requirements, high quantum efficiency, and insensitivity to magnetic fields, and they are relatively less expensive (103). To some extent, SiPMs provide an excellent alternative solution to traditional PMT detectors. Meanwhile, the development of some photon number resolving (PNR) detectors, such as supercon-ducting nanowire single-photon detectors (104) and transition edge sensors (105), has greatly improved the negative impact on experiments caused by the lack of PNR capability in non-PNR detectors (e.g., SiPM). Additionally, the development of charge-coupled device (CCD) cameras based on semiconductor materials, including electron-multiplying CCD (106), has allowed detectors to amplify signals on the chip and bypass readout noise to maintain high sensitivity at high speeds. This effectively enables real-time imaging, particularly the visualization of the structure of target tissues through real-time fluorescence intensity signals, and has become one of the most commonly used detectors in the FGS systems (107). Furthermore, the complementary metal-oxide-semiconductor can provide higher image acquisition speeds, lower power consumption, and is less costly to manufacture than CCD (77). In conclusion, semiconductor, PNR, and non-PNR detectors each have their advantages; the development of suitable fluorescence detectors is an important strategy to optimize the application of PDD and AFI in FGS.

Currently, the fluorescence signal acquisition and processing system mainly applies the fluorescence imaging and fluorescence spectroscopy methods. The former method collects AF images of different tissues through detectors and directly visualizes tumor cells based on the fluorescence images (108). This approach is simple, intuitive, has a large detection range, can diagnose in real-time, and does not require complex equipment, which is very favorable for clinical applications. Kriegmair et al. (70) used pseudocolor image processing to display composite autofluorescent images to differentiate between normal and cancerous tissues, achieving a diagnostic sensitivity and specificity of 96.7% and 53.8%, respectively. However, the fluorescence imaging method presents many disadvantages, such as the time-consuming image analysis, which is highly subjective, and the quality of the image being easily affected by the surrounding environment.

On the other hand, the fluorescence spectroscopy method is based on the difference in fluorescence intensity determined by collecting AF spectra from different tissues (109). Compared to the fluorescence imaging method, the fluorescence spectroscopy method is more rigorous and provides higher specificity as it does not need to be interpreted. Moreover, the spectral signals are rich in information, including various types of lesions, degree of tumor differentiation, and tissue subtypes (110). For example, Schuty et al. (111) analyzed the AF differences between melanoma, nevus, and normal skin by hyperspectral imaging and spectral vector analysis. The results indicated that spectral vector analysis has great potential for the diagnosis of melanoma. However, fluorescence spectroscopy also has some shortcomings, such as high equipment costs, complex structure, and the inability to visually display lesions. Moreover, this method is not as precise and convenient as fluorescence imaging in guiding surgical positioning. Therefore, combining fluorescence spectroscopy with fluorescence imaging may represent a potential approach to the FGS process.

Furthermore, in the field of FGS, the application of AI technology can enhance image recognition and provide surgical planning and decision making, ultimately achieving accuracy and safety in FGS (112). For example, Hardy et al. assessed the diagnostic efficacy of machine learning (ML) on regions of interest (ROI) in ICG-mediated FGS for 24 patients with colorectal liver metastases (CRLM). The results showed that the ML algorithms achieved a classification accuracy of 97.2% for CRLM ROIs (n = 132) within the 90s of ICG injection and all benign lesion ROIs (n = 6). The “Optimized Tree” classifier demonstrated an average accuracy of 97.2%, with a positive predictive value of 92.3% for benign lesions (113). Meanwhile, neural networks (NNs), as a branch of ML, mimics the structure of the human brain and extracts data features through hierarchical processing with self-learning and optimization capabilities. Compared with traditional statistical methods, NNs are superior in pattern recognition and calibration, reducing individual differences in fluorescent features (97). For example, convolutional neural networks (114) effectively process the fluorescence images generated by the FGS process through image processing, segmentation, feature extraction, and classification, and finally improves the accuracy and diagnostic efficacy of FGS. Finally, the trend of optimizing PDD and AFI by refining multiple parameters in fluorescence imaging systems will continue into the foreseeable future, as diagnostic and FGS applications are poised to reap significant benefits from the optimization strategy.

## 5 Conclusions

Fluorescence imaging, as an emerging technology for tumor diagnosis, facilitates timely detection and treatment of cancer. This review systematically describes the current research status and progress of PDD and AFI, highlighting the clinical application limitations of PDD and AFI for tumor diagnosis and FGS. The optimization of fluorescent probes, improvement of fluorescent imaging systems, and combined diagnostic strategies of PDD and AFI can largely improve their shortcomings in diagnosis and FGS. However, well-designed and substantial clinical studies are still needed to further substantiate this perspective. Further optimization introduces certain technical challenges to the application of PDD and AFI, such as the need to balance the optimal excitation wavelengths for PDD and AFI while reducing the signal-to-noise ratio, avoiding interference from ambient light in imaging, and optimization of AI-based fluorescent signal processing algorithms, etc. With further preclinical research and clinical trials, we believe that optimized PDD and AFI will be widely applied in clinical practice.
